# Intellectual-cultural orientation of family environment and adolescent depressive symptoms: the mediating role of game addiction

**DOI:** 10.3389/fpsyt.2026.1795262

**Published:** 2026-06-08

**Authors:** Yanli Ding, Yingyan Zhong, Xiaoya Cheng, Enzhao Cong, Jianhua Chen

**Affiliations:** 1 Shanghai Mental Health Center, Shanghai Jiao Tong University School of Medicine, Shanghai, China; 2 Shanghai Qingpu District Mental Health Center, Shanghai, China; 3 Shanghai Tenth People’s Hospital, School of Medicine, Tongji University, Shanghai, China; 4 Shanghai Institute of Traditional Chinese Medicine for Mental Health, Shanghai Clinical Research Center for Mental Health, Shanghai Key Laboratory of Mental Disorders Translational Research, Shanghai, China; 5 Brain Health Institute at National Center for Mental Disorder, Shanghai, China; 6 FuRong Laboratory, Changsha, Hunan, China

**Keywords:** adolescent, depressive symptoms, family environment, game addiction, intellectual-cultural orientation

## Abstract

**Introduction:**

Existing data indicate that an increasing number of adolescents are becoming addicted to online games, while the prevalence of depressive symptoms within this demographic is also on the rise. Depression is a primary comorbidity associated with game addiction, but the influencing factors and mechanisms remain unclear. This study aims to explore the mediating role of game addiction in the relationship between the intellectual-cultural orientation of family environment and adolescent depressive symptoms.

**Methods:**

A cross-sectional study was conducted on 1,105 pairs of mothers and adolescents in a high school in Henan Province, China, through online investigation from November 17, 2021 to December 11, 2021. The intellectual-cultural orientation, game addiction and adolescent depressive symptoms were measured by the subscale of Intellectual-cultural orientation in Family Environment Scale, Game Addiction Scale for Adolescents and Children’s Depression Inventory respectively. The SPSS PROCESS macro 3.3 software was used to analyze the mediating effect.

**Results:**

The findings revealed that Intellectual-cultural orientation was negatively correlated with both adolescent game addiction and depressive symptoms. Game addiction served as a significant mediator between Intellectual-cultural orientation and depressive symptoms in adolescents. Furthermore, gender and annual household income significantly associated with the strength of the mediating effect of game addiction on the relationship between Intellectual-cultural orientation and adolescent depressive symptoms. Specifically, boys and adolescents from low-incomefamilies were more likely to suffer from game addiction.

**Discussion:**

These findings suggest that future family based interventions aimed at preventing adolescent depression should specifically target the reduction of game time, particularly among boys and adolescents from low-income families.

## 1 Introduction

Early adolescence constitutes a critical neurodevelopmental period characterized by heightened vulnerability to the onset of psychopathology, particularly depressive symptomatology, which represents the most prevalent mental health concern in this population (1). In recent years, a maladaptive coping pattern has been observed among adolescents experiencing depressive symptoms: they tend to withdraw from real-world social and achievement-related activities, turning instead to digital escapism through excessive internet use as a means of regulating negative emotions (2–4). A substantial body of evidence indicates that depressive symptoms are a significant comorbidity associated with game addiction (5–7). Recent national data reveals 13.9% of China’s 1.079 billion internet users are adolescents aged 10-19 (8), with 33.4% demonstrating problematic mobile device dependence characterized by self-reported distress when separated from devices (9). This trend toward digital immersion coincides with an increasing prevalence of depressive symptoms (10), particularly among those meeting criteria for game disorder (11). Furthermore, adolescents with game addiction are at elevated risk for depressive symptoms, self-injury and suicide (12–15). Excessive gaming is associated with social isolation (16, 17), family conflict (18, 19), academic stress (20), and other health risks (21, 22). In the context of mounting environmental stressors, such adolescents may develop negative cognitive patterns, thereby increasing their susceptibility to clinical depression or exacerbating pre-existing depressive symptoms (23). While the co-occurrence of depressive symptoms and game addiction disorder is well-documented, the psychosocial mechanisms underlying their comorbidity remain insufficiently understood. Therefore, it is crucial to further investigate the factors influencing the comorbidity of depressive symptoms and game addiction disorder among adolescents.

Ecological systems theory posits that adolescent development is influenced by multiple interconnected environmental layers, with the family being a key proximal system (24) and the primary developmental context during adolescence (25, 26), exerting profound influences on social adaptation, psychological well-being, and personality development (27). Within this framework, a family’s intellectual-cultural orientation (ICO), the extent of its engagement in political, intellectual, and cultural discourse and shared learning (28), constitutes a core dimension of familial cultural capital. ICO facilitates adaptive lifestyle choices, strengthens stress resilience and problem-solving capacities (29). As a core feature of familial cultural capital (28), ICO demonstrates robust associations with cognitive maturation and prosocial skill development. Conversely, empirical evidence indicates that deficient ICO exposure is inversely related to adolescent psychopathology (30), and correlates with maladaptive outcomes, such as impaired self-concept, social competence deficits, and emotional dysregulation, which are risk factors for addictive behavioral patterns (31). From a cognitive-behavioral perspective, a family environment low in intellectual and cultural stimulation may fail to foster adaptive cognitive schemas and coping skills, potentially increasing the likelihood of maladaptive escapist behaviors like excessive gaming, which in turn may contribute to depressive affect. Recent Chinese researches continue to underscore the significant association between family factors, such as parental educational involvement and psychological control, and adolescent depressive symptoms, with cultural background playing a moderating role (32). The family atmosphere emerges as a decisive determinant of adolescent digital engagement patterns (22), with cohesive family atmospheres demonstrating protective effects against high-risk behaviors and pathological gaming (33). Additionally, other studies have shown that families with game addiction often exhibit low levels of intellectual and cultural orientation (34). While previous studies have explored the links between family environment, adolescent psychopathology, and addictive behaviors, research simultaneously examining the interplay between family intellectual-cultural orientation, game addiction, and adolescent depressive symptoms remains limited. Specifically, the potential mediating role of game addiction in the ICO-depression link, and how demographic factors like gender and income may influence this pathway, are areas that have received less attention.

Previous research has underscored the roles of gender (35) and annual household income (36) as important psychosocial moderators. Males are generally more likely to develop online game addiction (37), while females report higher rates of depressive symptoms (38). The impact of annual household income on family functioning is also evident. Research has demonstrated that economic hardship can impair parental mental health and reduce their capacity to provide warm and sensitive parenting (39), thereby limiting positive parent–child interactions (40) and constraining opportunities for intellectual and cultural enrichment (41). However, few studies have systematically examined how gender and income interact with family ICO, game addiction, and depressive symptoms in adolescents.

Many of the studies mentioned above have investigated the psychological and social factors associated with adolescent depressive symptoms, family culture, and game addiction. However, to the best of our knowledge, only limited research to date has examined the relationship among all three variables within a single study, potentially overlooking important insights regarding their interactions. Therefore, it is essential to conduct a study that explores the relationships and interactions between family culture, adolescent depressive symptoms, and game addiction. Additionally, it remains unclear how factors such as gender and annual household income influence and interact with family culture, adolescent depressive symptoms, and game addiction.

This study aimed to investigate the relationship among the intellectual-cultural orientation of the family environment, game addiction, and depressive symptoms in adolescents. We hypothesized that (1) ICO is negatively correlated with both game addiction and depressive symptoms in adolescents; (2) game addiction acts as a mediating factor between ICO and depressive symptoms in adolescents; and (3) gender and household income may moderate the mediating effect of game addiction on the relationship between ICO and depressive symptoms in adolescents.

## 2 Methods

### 2.1 Participants and procedure

This investigation employed a cross-sectional design to examine the relationships between family intellectual-cultural orientation, adolescent game addiction, and depressive symptoms. The study was conducted from November 17 to December 11, 2021, at a high school in Henan Province, China. A whole cluster sampling method was utilized to recruit participants, which included 2,487 students and 1,553 of their mothers. Data collection was administered online via a digital platform by school psychologists and class teachers. The data collection protocol commenced only after the informed consent process was completed. Eligibility to commence the questionnaire was contingent upon selecting “Agree”; selecting “Disagree” resulted in immediate termination of the session. Researchers emphasized to adolescent participants that all responses would be handled confidentially. Meanwhile, mothers were provided with a comprehensive debriefing regarding the study’s aims before their informed consent was secured. After completing the questionnaire, feedback on the research findings was provided to the school psychologists. Furthermore, researchers are dedicated to assisting them in implementing essential interventions for adolescents within relevant psychology courses.

To ensure data quality, rigorous validation checks were performed. Duplicate submissions (*N* = 55) were identified and removed based on IP addresses. Additionally, responses with completion times exceeding 3 standard deviations from the mean (N = 61) or with an average time of less than 2 seconds per item (*N* = 154) were deemed invalid and excluded (42). Data from mothers reporting an age under 25 or over 100 years (*N* = 2) and from adolescents outside the 14–18 age range (*N* = 36) were also discarded.

After these exclusions, the final analytical sample comprised 1,452 mothers (valid response rate: 93.50%) and 2,282 adolescents (valid response rate: 91.76%). For the dyadic analysis central to this study, successful matching of student identifiers with their mothers’ contact details yielded 1,105 valid mother-adolescent pairs. All personal identifiers were anonymized upon data collection, with each dyad assigned a unique code to protect privacy. The study received ethical approval from the Ethics Committee of Shanghai Mental Health Center (Ethics Approval Number: 2021-11). Common method bias was assessed using Harman’s single-factor test. The unrotated factor solution revealed that the first factor accounted for 24.45% of the total variance, which is below the critical threshold of 40%, suggesting that common method bias was not a major concern in this study.

### 2.2 Measures

#### 2.2.1 Intellectual-cultural orientation

Intellectual-cultural orientation was assessed by the subscale of Intellectual- cultural orientation in Family Environment Scale (FES) (43), as reported by mothers. The scale comprises 9 items. Responses were recorded dichotomously, with each affirmative response scored as 1 point and each negative response scored as 2 points. The calculation of the total score follows the methodology outlined in the literature that guided the development of the scale (43). A higher total score indicates a greater level of intellectual-cultural orientation within the family environment. In the present sample, the subscale demonstrated a Cronbach’s α of 0.533. The observed α coefficient is below the conventional threshold of 0.7, indicating suboptimal internal consistency in this specific sample. This is acknowledged as a limitation of the present study. However, this result is consistent with the reliability and validity test of the scale in the context of adolescent population in Chinese culture (44), and its potential implications are discussed in detail in section 4. We conducted additional item-level analyses to further examine the scale’s performance in our sample (see **Supplementary Table 1** in **Supplementary Material**). The corrected item-total correlations (CITC) ranged from 0.029 to 0.391, but the deletion of any single item did not increase the Cronbach’s α coefficient above 0.6, indicating that the suboptimal internal consistency was not attributable to a few poorly performing items but rather a characteristic of the scale’s measurement in this specific, homogeneous sample.

#### 2.2.2 Game addiction

Game addiction was assessed using the 21-item Game Addiction Scale for Adolescents (GAS) (45), as reported by adolescents. The scale comprises 21 items, each assessed on a 5-point scale (1-5). A higher total score reflects a more severe level of game addiction. For the abbreviated 7-item version, addiction is indicated if more than half of the responses are rated ‘Sometimes’ (3 points) or higher. The scale showed excellent internal consistency in this study, with a Cronbach’s α of 0.964.

#### 2.2.3 Adolescent depressive symptoms

The Children’s Depression Inventory (CDI) was used to measure depressive symptoms (46), as reported by adolescents. This 27-item instrument, designed for youths aged 14-18, employs a 3-point scale (0-2). A total score of 19 or above is considered clinically significant. Higher scores on the CDI indicate greater severity of depressive symptoms. The measure achieved good reliability in our sample, with a Cronbach’s α of 0.874.

### 2.3 Statistical analysis

Data analyses were performed using SPSS Statistics version 25.0. Descriptive statistics are presented as mean (*M*) ± standard (*SD*) or frequency (percentage). Consider that the scale scores involve rank variables, Spearman’s correlation analysis was conducted to examine the bivariate associations among the primary variables (intellectual-cultural orientation, game addiction, depressive symptoms). Group differences in these variables across gender and annual household income were assessed using independent-samples *t* tests.

The hypothesized mediating effect of game addiction on the relationship between intellectual-cultural orientation and depressive symptoms was tested using Model 4 within the SPSS PROCESS macro (version 3.3), with 5,000 bootstrap samples for bias-corrected confidence intervals. Adolescent age and parental education level were included as control variables in the mediation model. The mediation effect was considered statistically significant if the 95% confidence interval (CI) for the indirect effect did not include zero. To explore moderation, the analysis was conducted separately by gender and annual household income (using 100,000 yuan as a cutoff). Furthermore, we also utilized Model 59 within the SPSS PROCESS macro (version 3.3) to verify whether the observed differences between males and females or across income groups are statistically significant. The threshold for statistical significance was set at *p* < 0.05 for all analyses. Standardized regression coefficients, bias-corrected bootstrap confidence intervals, and effect sizes (for subgroup comparisons) are reported where applicable. The assumptions of the statistical tests (e.g., normality of residuals, homogeneity of variance) were checked, and no substantial violations were observed. Multicollinearity diagnostics (Variance Inflation Factor, VIF) were conducted for regression models, with all VIF values below 2, indicating no concerning multicollinearity.

## 3 Result

### 3.1 Demographic and descriptive analysis

Out of 1,105 pairs of valid questionnaires, the prevalence of depressive symptoms in adolescents was 21.27%, among whom the rate of game addiction was 43.40%. The prevalence of game addiction in adolescents was 29.32%, among whom 31.48% exhibited depressive symptoms. The prevalence of game addiction was 71.60% (232/324) in males and 28.40% (92/324) in females. The comorbidity rate of depressive symptoms and game addiction among the adolescents was 9.23%. Most mothers were younger than 45 (97.74%, *M* (age) = 34.05, *SD* = 4.89). The sample of adolescents was aged from 14 to 18 years (*M* (age) = 16.54, *SD* = 0.87). Among them, there were 557 (50.41%) boys and 548 (49.59%) girls. There were 350 adolescents with an annual household income of less than 20,000 yuan, 400 within the 20,000 to 50,000 yuan, 224 within the 50,000 to 100,000 yuan, and 95 within the 100,000 to 150,000 yuan. Additionally, there were 36 adolescents with annual incomes exceeding 150,000 yuan. Hence, there were 131 (11.86%) adolescents from high- income families, defined as those with an annual household income of 100,000 yuan or more. There were 974 (88.14%) adolescents from low-income families, defined as those with an annual household income of less than 100,000 yuan. **Table 1** shows the descriptive statistics and scores of the intellectual-cultural orientation, game addiction and depressive symptoms in adolescents.

**Table 1:** Descriptive statistics and scores of the intellectual-cultural orientation, game addiction and depressive symptoms.

Variables	*M* ± *SD*/*n* (%)
**Mother’s age (years)**	34.05 ± 4.89
**Adolescent’s age (years)**	16.54 ± 0.87
Gender	
Boys	557 (50.41)
Girls	548 (49.59)
Annual household income	
Less than 20,000 yuan	350 (31.67)
20,000 to 50,000 yuan	400 (36.20)
50,000 to 100,000 yuan	224 (20.27)
100,000 to 150,000 yuan	95 (8.60)
More than 150,000 yuan	36 (3.26)
Father’s education level	
Graduate degree or above	6 (0.54)
Undergraduate	75 (6.79)
Senior school or Technical secondary school	343 (31.04)
Junior school and below	681 (61.63)
Mother’s education level	
Graduate degree or above	5 (0.45)
Undergraduate	97 (8.78)
Senior school or Technical secondary school	297 (26.88)
Junior school and below	706 (63.89)
**Intellectual-cultural orientation**	4.50 ± 1.88
**Game addiction**	37.14 ± 16.09
**Depressive symptoms**	13.71 ± 7.14

The bold values indicate the key variables involved in the subsequent mediation model.

### 3.2 Correlation analysis

Correlations among intellectual-cultural orientation, game addiction and depressive symptoms were assessed by calculating the Spearman correlation coefficient (see **Table 2**). As was hypothesized, intellectual-cultural orientation was weakly but significantly negatively associated with game addiction (*r* = −0.09, *p* < 0.01) and depressive symptoms (*r* = −0.11, *p* < 0.001). Game addiction was weakly but significantly positively associated with depressive symptoms (*r* = 0.26, *p* < 0.001).

**Table 2:** Correlations among intellectual-cultural orientation, game addiction and depressive symptoms.

Variables	1	2	3
1. Intellectual-cultural orientation	−		
2. Game addiction	−0.09^**^	−	
3. Depressive symptoms	−0.11^***^	0.26^***^	−

^**^ *p* < 0.01, ^***^p < 0.001

### 3.3 Differential analysis

As depicted in **Table 3**, the scores of game addiction were significantly higher in boys compared to girls (*t*1103 = 11.326, *p* < 0.001, *d* = 0.681). Meanwhile, the scores of game addiction in adolescents from low-income families were significantly higher than ones from high-income families (*t*1103 = 2.006, *p* < 0.05, *d* = 0.196). There were no significant differences observed in intellectual-cultural orientation and depressive symptoms across gender and annual household income. Therefore, the mediating effect of game addiction across different genders and household income merits further statistical analysis.

**Table 3:** Differences in intellectual-cultural orientation, game addiction and depressive symptoms across gender and annual household income.

Variables	Gender/income	*M* ± *SD*	*t*_1103_	*p*	*Cohen’s d*
Intellectual-cultural orientation	Boys	4.46 ± 1.93	0.650	0.516	0.043
	Girls	4.54 ± 1.82			
Game addiction	Boys	42.29 ± 17.03	11.326^***^	<0.001	0.681
	Girls	31.91 ± 13.16			
Depressive symptoms	Boys	13.45 ± 7.36	1.225	0.221	0.074
	Girls	13.98 ± 6.89			
Intellectual-cultural orientation	High income	4.53 ± 1.95	0.177	0.860	0.015
	Low income	4.50 ± 1.87			
Game addiction	High income	34.50 ± 15.14	2.006^*^	0.045	0.196
	Low income	37.49 ± 16.18			
Depressive symptoms	High income	13.05 ± 7.24	1.143	0.253	0.104
	Low income	13.80 ± 7.12			

^*^ *p* < 0.05, ^***^p < 0.001

### 3.4 Mediating effect analysis

**Figure 1:**
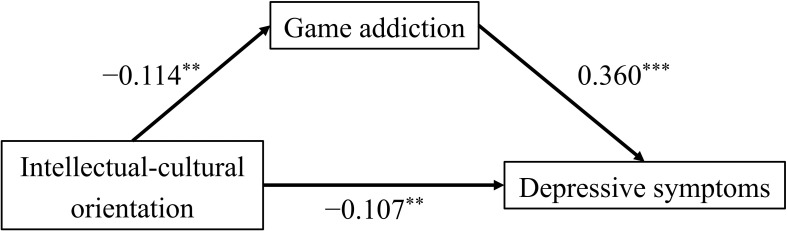
The mediation model for intellectual-cultural orientation, game addiction and depressive symptoms in boys. ^**^p < 0.01, ^***^p < 0.001.

Intellectual-cultural orientation, game addiction and depressive symptoms in adolescents were significantly correlated, meeting the requirements for further mediation analysis (47). As depicted in **Table 4**, for all adolescents included in this study, Model A indicated a significant mediating effect of game addiction between intellectual-cultural orientation and depressive symptoms (effect value: −0.106, 95%CI = [−0.183, −0.038]) (see **Supplementary Figure 1** in **Supplementary Material**). For boys, Model B showed that game addiction played a significant mediating role between intellectual-cultural orientation and depressive symptoms (effect value: −0.157, 95%CI= [−0.286, −0.045]), with an effect size of 27.89%. In Model B, intellectual-cultural orientation negatively was negatively associated with game addiction (*β* = −0.114, *p* < 0.01) and depressive symptoms (*β* = −0.107, *p* < 0.01), while game addiction positively was positively associated with depressive symptoms in boys (*β* = 0.360, *p* < 0.001) (see **Figure 1**). However, for girls, Model C revealed that game addiction did not serve as a mediating factor between intellectual-cultural orientation and depressive symptoms (effect value: −0.063, 95%CI = [−0.154, 0.018]) (see **Supplementary Figure 2** in **Supplementary Material**). Further model analysis in Model 59 within the SPSS PROCESS macro showed that the coefficients of the interaction terms between gender and Intellectual-cultural (coefficient: 0.324, 95%CI = [−0.101, 0.750]), as well as gender and game addiction were not statistically significant (coefficient: −0.007, 95%CI = [−0.061, 0.048]).

**Table 4:** Bootstrap analysis of the significance test of the mediating effect.

Path	Effect	Effect size (%)	*SE*	Bias-corrected 95% CI	
				Lower	Upper
Model A (All)					
Total effects	−0.350		0.115	−0.575	−0.125
Direct effects	−0.245	69.80	0.110	−0.461	−0.029
Intellectual-cultural orientation → Game addiction → Depressive symptoms	−0.106	30.20	0.037	−0.183	−0.038
Model B (Boys)					
Total effects	−0.563		0.161	−0.880	−0.247
Direct effects	−0.406	72.11	0.152	−0.704	−0.108
Intellectual-cultural orientation → Game addiction → Depressive symptoms	−0.157	27.89	0.062	−0.286	−0.045
Model C (Girls)					
Total effects	−0.109		0.163	−0.429	0.211
Direct effects	−0.046	42.20	0.157	−0.354	0.262
Intellectual-cultural orientation → Game addiction → Depressive symptoms	−0.063	57.80	0.044	−0.154	0.018
Model D (Low income)					
Total effects	−0.372		0.123	−0.613	−0.131
Direct effects	−0.243	65.32	0.118	−0.475	−0.011
Intellectual-cultural orientation → Game addiction → Depressive symptoms	−0.129	34.68	0.041	−0.217	−0.056
Model E (High income)					
Total effects	−0.185		0.326	−0.830	0.459
Direct effects	−0.239	128.64	0.314	−0.860	0.383
Intellectual-cultural orientation → Game addiction → Depressive symptoms	0.053	−28.64	0.094	−0.128	0.252

CI, confidence interval; effect size, %: the ratio of the effect to total effect; *SE*, standard error.

**Figure 2:**
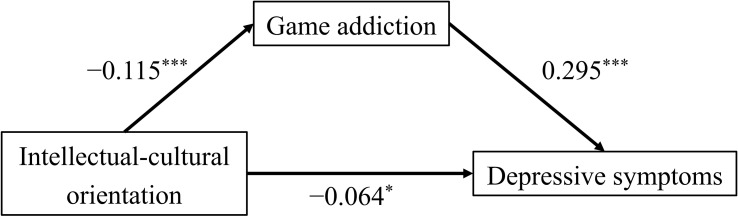
The mediation model for intellectual-cultural orientation, game addiction and depressive symptoms in adolescents from low-income families. ^*^p < 0.05, ^***^p < 0.001.

In addition, for adolescents from low-income families, Model D showed that game addiction played a significant mediating role between intellectual-cultural orientation and depressive symptoms (effect value: −0.129, 95%CI = [−0.217, −0.056]), with an effect size of 34.68%. In Model D, intellectual-cultural orientation was negatively associated with game addiction (*β* = −0.115, *p* < 0.001) and depressive symptoms (*β* = −0.064, *p* < 0.05), while game addiction was positively associated with depressive symptoms in adolescents from low-income families (*β* = 0.295, *p* < 0.001) (see **Figure 2**). However, for adolescents from high-income families, Model E revealed that game addiction did not serve as a mediating factor between intellectual-cultural orientation and depressive symptoms [effect value: −0.053, 95%CI = (−0.128, 0.252)] (see **Supplementary Figure 3** in **Supplementary Material**). Further model analysis in Model 59 within the SPSS PROCESS macro showed that the coefficients of the interaction terms between income and Intellectual-cultural [coefficient: −0.097, 95%CI = (−0.740, 0.546)], as well as income and game addiction were not statistically significant [coefficient: 0.012, 95%CI = (−0.070, 0.093)].

## 4 Discussion

The present study investigated the complex interplay between family intellectual-cultural orientation (ICO), adolescent game addiction, and depressive symptoms, with a specific focus on the mediating role of game addiction and the moderating effects of gender and annual household income. The results indicate that a higher level of ICO within the family environment is associated with lower levels of both game addiction and depressive symptoms among adolescents. Furthermore, game addiction was identified as a significant mediator in the relationship between ICO and depressive symptoms. Importantly, this mediating pathway was found to be present specifically for boys and for adolescents from low-income families, but not for girls or those from high-income households, suggesting that gender and socioeconomic status are associated with the strength of this indirect association.

Among the 1,105 pairs of valid questionnaires in this study, the prevalence of depressive symptoms among adolescents was 21.27%. This rate is marginally lower than the approximate 24% average documented in recent literature (48). The prevalence of game addiction was 29.32%, exceeding the rates documented in prior studies (49, 50). Besides, a notable bidirectional comorbidity was observed: nearly one-third (31.48%) of adolescents with game addiction had comorbid depressive symptoms, and an even higher proportion (43.40%) of those with depressive symptoms were diagnosed with game addiction, suggesting depressive symptoms are a significant risk factor for game addiction. Numerous studies have shown that adolescents experiencing depressive symptoms may turn to the Internet to fulfill their needs for interpersonal relationships and escapism (51), a behavior reinforced by the positive affect associated with online use. This can lead to the use of games as a maladaptive coping strategy to alleviate stress and seek transient gratification. Conversely, once established, game addiction is characterized by dysfunctional coping strategies (e.g., denial, behavioral disengagement) (52), which can exacerbate depressive symptomatology (53), creating a cyclical relationship. Therefore, for adolescents with depressive symptoms, it is crucial to monitor their daily activities, pay attention to their gaming time, adjust their lifestyles accordingly, and address any negative cognitions and behaviors.

This study further elucidates that the intellectual and cultural orientation of the family environment is indirectly associated with adolescent depressive symptoms through the mediating pathway of game addiction. According to Maslow’s hierarchy of needs, once basic physiological and safety needs are met, the pursuit of higher-level motivations is central to adolescent development and is significantly shaped by the family climate (22). Families characterized by less active and engaged parenting are more likely to exhibit poorer overall functioning, thereby increasing the vulnerability of adolescents to depressive disorders (23). When adolescents perceive a lack of familial support and intellectual stimulation, they may seek alternative avenues for fulfillment, such as virtual relationships and online achievements (54). These digital spaces can provide transient feelings of intimacy, belonging, and competence (55), thereby increasing the risk of compulsive gaming (22). Conversely, exposure to socio-cultural and intellectual activities within the family fosters adolescent subjective well-being and may buffer against the onset of depressive episodes (28). Empirical evidence consistently identifies low ICO as a robust familial predictor of game addiction (56). Consistent with these findings, our study confirms the negative correlations between ICO and both game addiction and depressive symptoms, and establishes game addiction as a partial mediator. The indirect effect, though significant, was small, which may reflect the distal nature of family cultural orientation as an influence on individual-level psychological outcomes, operating through multiple intermediate pathways.​ Therefore, fostering a family environment rich in intellectual and cultural exchanges is essential. Parents are encouraged to dedicate quality time to engaging in mutually satisfying and intellectually stimulating activities with their adolescents. Furthermore, the observed stronger mediating effect of game addiction in low-income families may be partly explained by the relative scarcity of alternative cultural and recreational resources, as well as potential digital divides in parenting practices, which aligns with recent research on family cultural capital and digital media use in Chinese contexts (57).

Consistent with most previous studies (37), the prevalence of game addiction is higher in men than in women. Among adolescents with game addiction in our sample, boys accounted for 71.60%, while girls accounted for 28.40%. Moreover, game addiction played a significant mediating role between intellectual-cultural orientation and depressive symptoms only for boys. These data suggest that the prevalence of game addiction is indeed higher among males in this study, which aligns with the findings of previous research. Notably, among individuals with game addiction, men exhibit lower self-esteem compared to women (58). Previous research indicates that as people become more isolated and depressed, they tend to immerse themselves further in the Internet. Both loneliness and lack of social support aggravate depressive symptoms in adolescents (59). Boys who lack confidence in their social lives are particularly susceptible to this trend (60). Additionally, other studies have shown that male students often use the Internet as a means of leisure and entertainment, typically accompanied by positive emotions. This can lead to a cycle of game addiction, where longer gaming sessions correlate with increased feelings of happiness (61). Furthermore, Shek and Yu found that boys tend to be enthusiastic about the development of Internet technology and learning about Internet applications (33). This suggests that men with game addiction are more likely to become addicted to online games than women, which can result in negative consequences. Therefore, it is crucial to pay closer attention to the mental health of young men struggling with game addiction.

For adolescents from low-income families, our study demonstrates that game addiction significantly mediates the relationship between intellectual and cultural orientation and depressive symptoms. In China, education serves as a crucial asset for securing important jobs in a competitive labor market and is a vital means of improving one’s quality of life, particularly for those from families with lower socioeconomic status (62). However, due to the economic challenges faced by low-income families, parents often allocate most of their financial resources to necessities and the education of their teenagers, leaving little to invest in the intellectual and social development of their children (36). This results in a reduced likelihood of adolescents accessing cognitively stimulating materials, such as books and toys, as well as enriching social experiences, including cultural events and activities (41). Additionally, low-income families frequently experience insufficient resources, limited shared decision-making and parenting responsibilities, a lack of mutual support and guidance, and inadequate emotional care, along with insufficient time investment in family relationships (22). Consequently, adolescents from low-income families often seek to fulfill their psychological needs through online games (63). Parents from low-income families often have limited educational backgrounds, which may result in a lack of awareness regarding the negative effects of game addiction and the strategies to prevent it (22). This lack of knowledge can contribute to the prevalence of game addiction among adolescents from families with low annual incomes. Therefore, it is essential to enhance social and school support systems in low-income communities to provide greater psychological and behavioral assistance to young people.

The limitations of this study should be acknowledged. Firstly, the cross-sectional design limits the ability to examine long-term changes in adolescent family environment and to draw causal conclusions regarding the relationships between ICO, game addiction, and depressive symptoms. Notably, the relationships modeled could be bidirectional; for instance, depressive symptoms may both contribute to and result from excessive gaming, and may also influence family interactions and the perceived intellectual-cultural climate. The associations observed in this study warrant further investigation through longitudinal or experimental designs. Secondly, the internal consistency (Cronbach’s α = 0.533) of the ICO subscale in this sample was suboptimal, indicating a measurement limitation. The modest alpha may be attributable to the scale’s dichotomous response format, the relative homogeneity of our sample (predominantly mothers with lower education and families with modest incomes), and potential context effects of online data collection. This measurement error may have attenuated the observed effect sizes of mediation. Thirdly, the sample comprised high school students from a single Chinese province, which may limit the generalizability of the findings to other regions with different socioeconomic or cultural contexts. Future studies with more diverse and representative samples are needed to enhance external validity. Fourthly, the non-significant interaction terms in these exploratory models may reflect limited statistical power in subgroup analyses or more complex underlying mechanisms. Future researches should employ formal moderated mediation models that include interaction terms (e.g., PROCESS Model 7, 14, 59) with larger samples to verify and extend these findings. Fifthly, another limitation involves potentially insufficient control for confounding variables. While adolescent age and parental education were adjusted for, other relevant factors, such as adolescent screen time, sleep duration, parental psychopathology, family structure, academic stress, and urban versus rural residence, were not accounted for, which may leave residual confounding. Finally, a limitation of this study is its reliance exclusively on self-report and maternal-report questionnaires for assessment. The perspective of fathers was notably absent. Future research would benefit from incorporating multi-informant reports, including both subjective and objective measures from a wider range of family members.

## 5 Conclusion

In summary, this study found that intellectual cultural orientation was negatively correlated with game addiction and depressive symptoms in adolescents. Additionally, game addiction served as a significant mediator between intellectual cultural orientation and depressive symptoms in this population. Our research also indicates that gender and family income have substantial moderating effects on the mediating role of game addiction in the relationship between​ intellectual cultural orientation and adolescent depressive symptoms. Specifically, male adolescents and those from low-income families are important predictors of game addiction. Consequently, it is evident that adolescents experiencing depressive symptoms are more likely to exhibit maladaptive behaviors, such as game addiction. Therefore, it is crucial to focus on enhancing the family environment and cultural support. In particular, for families with boys, educators and parents should address gender differences in game addiction and provide appropriate counseling and support to meet the needs of males during adolescence. Parents of teenagers struggling with game addiction can take several steps to help reduce their children’s dependence on gaming. First, they can enhance teenagers’ understanding of game usage, fostering critical thinking about their relationship with the Internet and mitigating ineffective coping strategies related to academic and life pressures. Second, behavioral interventions can be implemented to some extent, such as setting limits on gaming time and allowing gameplay only during designated hours. Furthermore, parents can acknowledge the financial needs of their teenagers by providing an appropriate monetary allowance and guiding them in the responsible use of money. Finally, improving the family living environment is essential. Establishing a positive family atmosphere, increasing quality time spent together, and fostering open communication can create a supportive environment for teaching and enjoyable interactions. For families facing economic challenges, it is vital to leverage social and school resources. Through community and educational support, we can further assist adolescents from low-income families in enhancing their psychological well-being and addressing maladaptive behavior patterns.

## Data Availability

The raw data supporting the conclusions of this article will be made available by the authors, without undue reservation.
