# Supplementary material for: Intellectual-cultural orientation of family environment and adolescent depressive symptoms: the mediating role of game addiction
Source: Front Psychiatry. 2026 Jun 8;17:1795262. doi: 10.3389/fpsyt.2026.1795262 (PMC13283969; doi:10.3389/fpsyt.2026.1795262)
Supplement: Supplementary file 1 [file SupplementaryFile1.docx]

**Supplementary Information**

**Table S1.** Item-total statistics of the subscale of Intellectual-cultural orientation in Family Environment Scale

| Items | Corrected Item-Total Correlation | Cronbach’s α if Item Deleted |
| --- | --- | --- |
| 1 | 0.309 | 0.480 |
| 2 | 0.235 | 0.506 |
| 3 | 0.031 | 0.559 |
| 4 | 0.208 | 0.514 |
| 5 | 0.303 | 0.481 |
| 6 | 0.246 | 0.502 |
| 7 | 0.359 | 0.462 |
| 8 | 0.029 | 0.558 |
| 9 | 0.391 | 0.449 |


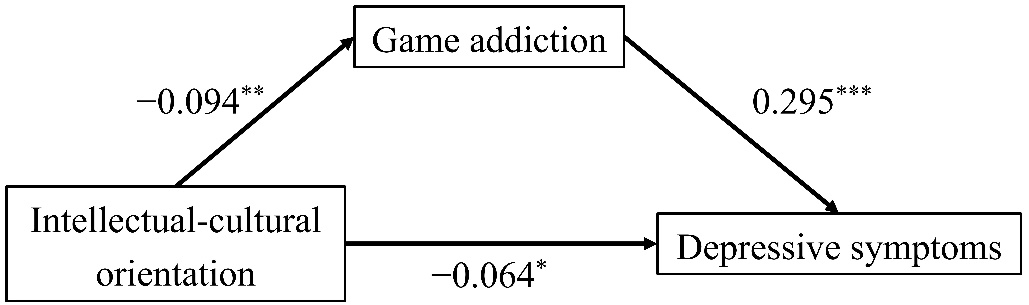


**Figure S1.** The mediation model for intellectual-cultural orientation, game addiction and depressive symptoms in all adolescents. ^**^*p* < 0.01, ^***^*p* < 0.001


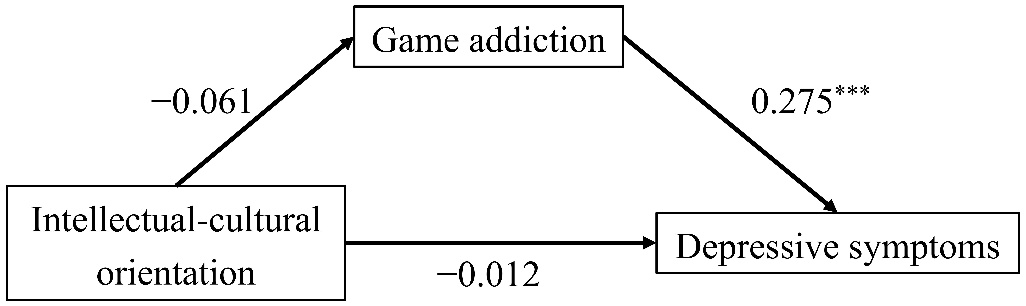


**Figure S2.** The mediation model for intellectual-cultural orientation, game addiction and depressive symptoms in girls. ^**^*p* < 0.01, ^***^*p* < 0.001


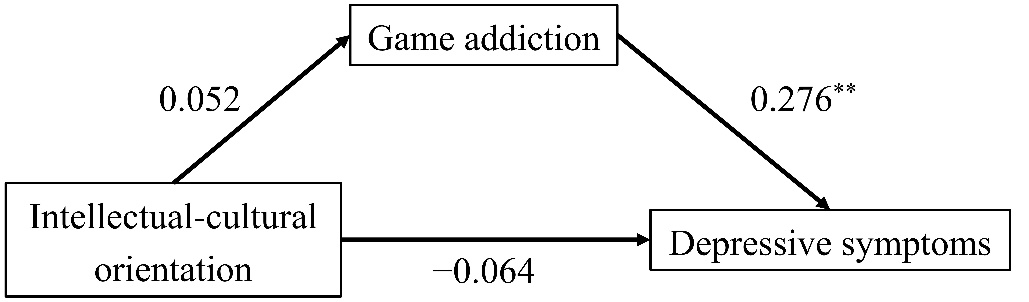


**Figure S3.** The mediation model for intellectual-cultural orientation, game addiction and depressive symptoms in adolescents from high-income families. ^**^*p* < 0.01, ^***^*p* < 0.001
